# Healthcare Transformation in the Post-Coronavirus Pandemic Era

**DOI:** 10.3389/fmed.2020.00429

**Published:** 2020-07-28

**Authors:** Abdul Rahman Jazieh, Zisis Kozlakidis

**Affiliations:** ^1^Department of Oncology, King Abdullah International Medical Research Center, King Saud bin Abdulaziz University for Health Sciences, Riyadh, Saudi Arabia; ^2^Laboratory Services and Biobanking, International Agency for Research on Cancer, World Health Organization, Lyon, France

**Keywords:** COVID-19, healthcare transformation, ethics, technological innovation, coronavirus

The effects of the coronavirus disease 2019 (COVID-19) pandemic globally are striking as it impacts greatly the social, political, economic, and healthcare aspects of many countries. The toll of this pandemic quantified with human lives and suffering (1), the psychosocial impact (2), and the economic slowdown (3) constitute strong reasons to translate experiences into actionable lessons, not simply to prevent similar future crises, but rather to improve the whole spectrum of population health and healthcare delivery. This is the third coronavirus (CoV) outbreak of international concern in 20 years, after the severe acute respiratory syndrome (SARS-CoV) and the Middle-East respiratory syndrome (MERS-CoV), in addition to other viral outbreaks such as Zika virus and Ebola virus over the last decade. It becomes clear that infectious diseases should be considered among the most important health hazards that we will need to continue facing in the foreseeable future (4). Thus, the transformation of various aspects at the individual as well as the societal and governmental levels seems inevitable.

The COVID-19 pandemic has become a reality check for many aspects of healthcare systems, especially regarding their overall readiness. Public health surveillance programs and available infrastructures were shown as not consistently optimal (5–7). Additionally, healthcare systems appeared unable to absorb and manage sudden and persistent pressures on their workload especially in the settings of acute care. Even though contingency plans were often in place, healthcare systems seemed unable to cope with the sudden, intense surge in demand (8, 9). From a policy perspective, potential delay(s) in committing to major decisions, such as lockdown measures, in an “epidemiologically timely fashion” could significantly impact downstream healthcare outcomes (10, 11). The latter is of particular importance, as healthcare challenges in one country should be considered both an internal and a potentially global challenge, at least for infectious diseases (12, 13). Finally, the speed at which a global public health issue translated into a financial downturn, affecting many different industries, was underestimated (3, 14).

The COVID-19 pandemic acts as a transformation catalyst, accelerating the implementation and adoption of changes in public health interventions. Thus, a new model of healthcare delivery emerges with more emphasis on preventive measures, remote care, and substantial technological dependence. However, these are juxtaposed against ongoing technical challenges to meet the surge capacity in laboratory testing, the fast-tracked implementation of new technologies, the mental health concerns, the ethical concerns on the potential rationing of insufficient resources, and the protection of privacy and personal data during times of crises. Taking the former into account, the following aspects seem likely to emerge as most affected in the post-COVID-19 era.

## Shifting Greater Patient Numbers To Remote Care

Remote care or telehealth services were already used in emergencies, crises, and routine care previously (15, 16). During the COVID-19 pandemic, their wider utilization has accelerated. Telehealth services have now been used in the large-scale screening of patients prior to their visit and triage assessment, in the routine monitoring of patients at home, for remote clinical encounters, or supervising patient care by off-site experts (17–19). It is likely that a significant portion of such services will remain telehealth-based post COVID-19, e.g., the remote monitoring and management of greater numbers of patients, as it provides higher convenience and better patient-centered care, thus partially addressing the healthcare system flow rate and capacity challenges.

This has been observed in mental healthcare as well, where the pandemic became a catalyst for the implementation of online therapy and e-health tools in routine practice, following more than two decades of many brilliant, but mostly failed, attempts (20, 21). Imperatives dominating the field, e.g., that “the clinician/patient therapeutic alliance can only be established face-to-face,” in spite of research showing the opposite (22), are being resolved. It is likely that once mental healthcare institutions have developed the capabilities post COVID-19 of serving their patients *via* different digital technologies, there is little reason for them to give all of these up, in view of the advantages they have experienced over an extended period of crisis response (23). A future “blended approach” is likely to emerge, where e-mental-health solutions occupy a greater part of routine services. Additionally, the currently developed expertise can be used in expanding a wider public e-mental-health approach, utilizing not only guided but also fully self-guided interventions, such as self-help apps or online therapeutic modules (24). The latter could also be tested and eventually applied in settings and countries with scarce mental health resources, where such need has been previously identified (25), as a positive post-COVID-19 long-term outcome.

This system evolution is likely to serve as an adjunct for the gradual adoption of further new technologies, for example, the use of drones as delivery vehicles for critical supplies, robotics, the widespread 3D-printing of healthcare-related items, and smartphone-enabled monitoring of patient adherence to treatments (26, 27).

## Improved Emphasis on Surveillance Systems and Data Analysis

The speed by which SARS-CoV-2 spread globally highlights once more that the need for reliable and representative surveillance systems for infectious diseases remains as acute as ever. Public health surveillance for infectious diseases uses reported positive results from sentinel clinical laboratories or laboratory networks to survey the presence of specific microbial agents that constitute a threat to public health in a given population (28). However, the continuing rationalization of public health costs has led to the consolidation of a number of clinical microbiology laboratories involving a shift toward laboratory amalgamation. Through this consolidation activity, an operational model emerged with large centralized clinical laboratories performing on one central platform and one or several distal laboratories dealing locally only with urgent analyses (29, 30). It would be informative to see if this reduction in the number of small clinical laboratories and the aggregation of the remaining ones conditioned or not the ability to detect epidemiological changes in the context of COVID-19.

Therefore, the routine use of big data and artificial intelligence approaches to model crises and to identify and understand the weaknesses of existing systems (close to real-time) would be necessary in order to strengthen existing structures. Mobile-enabled technologies can now be deployed *en masse* to monitor quarantined individuals and to trace exposed individuals in a timely and accurate fashion within regions and/or countries, as in the cases of South Korea and Taiwan (31). These are some of the new tools likely to move further into the public health sphere and support the understanding in an interconnected and hypercomplex global environment. The necessity for international collaboration and sharing of information between competent healthcare authorities during crises has been highlighted many times previously, as well as the rapid deployment of specialist teams on the ground, and this is likely to be strengthened even further post COVID-19 (32, 33).

Any such changes would need to be accompanied by a greater public awareness of the health systems, new and/or better tools, and their potential implementations in order to combat infectious disease outbreaks. As such, the interaction with social media and behavioral science is likely to be used extensively for health promotion, education, and mass communications (34). However, the pandemic has also highlighted that poor health literacy among the general population is an underestimated public health problem globally (35). Improving public health literacy is now essential as it might help people to grasp the reasons behind the recommendations and reflect on outcomes of their various possible actions, especially in the context of resource-restricted settings (36).

## Development of Legislative, Political, and Healthcare Management Systems

While the COVID-19 outbreak accelerated many of the above processes, there still remain challenges, including, for example, credentialing, licensing, reimbursement, and issues related to technology, security, privacy, safety, and litigations (37–39). More specifically, in the ethical field and from an individual perspective, the collection and availability of vast amounts of information regarding people (e.g., *via* geo-tagged social networks) makes full data anonymization ineffective in protecting the identity of the data source, making it only more difficult, yet still feasible *via* the use of advanced systems and triangulation, to (re)identify individuals (40). As such, the ethical imperative of transparency with regard to the dangers of downstream data linkage and inadvertent individual identification should be upheld (41). From a population-level perspective, if systems are designed to be entirely reliant on anonymous data in order to protect data contributors, they might not work very well-either, as the element of information accountability and, hence, transparency is affected. Especially in the case of humanitarian emergencies, and certainly communicable disease outbreaks, anonymous information is current best practice, but cannot be considered as the ethical panacea (42).

It should be noted that public health ethics differs from clinical ethics in that it requires giving priority to promoting the common good over protecting individual autonomy (43). This ethical contrast becomes even greater in resource-restricted settings during public health emergencies, where overwhelmed healthcare systems might instigate the rationing of staff and/or medical supplies, with distressing decision-making, such as who receives life support (44).

One of the defining aspects of the current pandemic was the unprecedented levels of misinformation, conspiracy theories, and rumors reproduced by lay and social media related to COVID-19; these can only be counterproductive in the fight against the current epidemic, both in the short and long term. Perhaps, this is an outcome of the pandemic taking place during the “social media age.” (45). The WHO responded to the “infodemic” releasing a statement and suppressing several such measures advocated online and in social media, which are not effective in the treatment of COVID-19, and has done so ongoingly (46). In terms of responses, social media platforms have responded to the majority of the social media posts rated false by fact-checkers by removing them or attaching various warnings. However, as the number of English-language fact-checks rose more than 900% from January to March, outpacing the available fact-checking resources, misinformation has almost certainly grown even faster (47). Consistency in the public health messaging as well as increased funding dedicated to fact-checking seems to be needed as the immediate first step.

**Figure 1:**
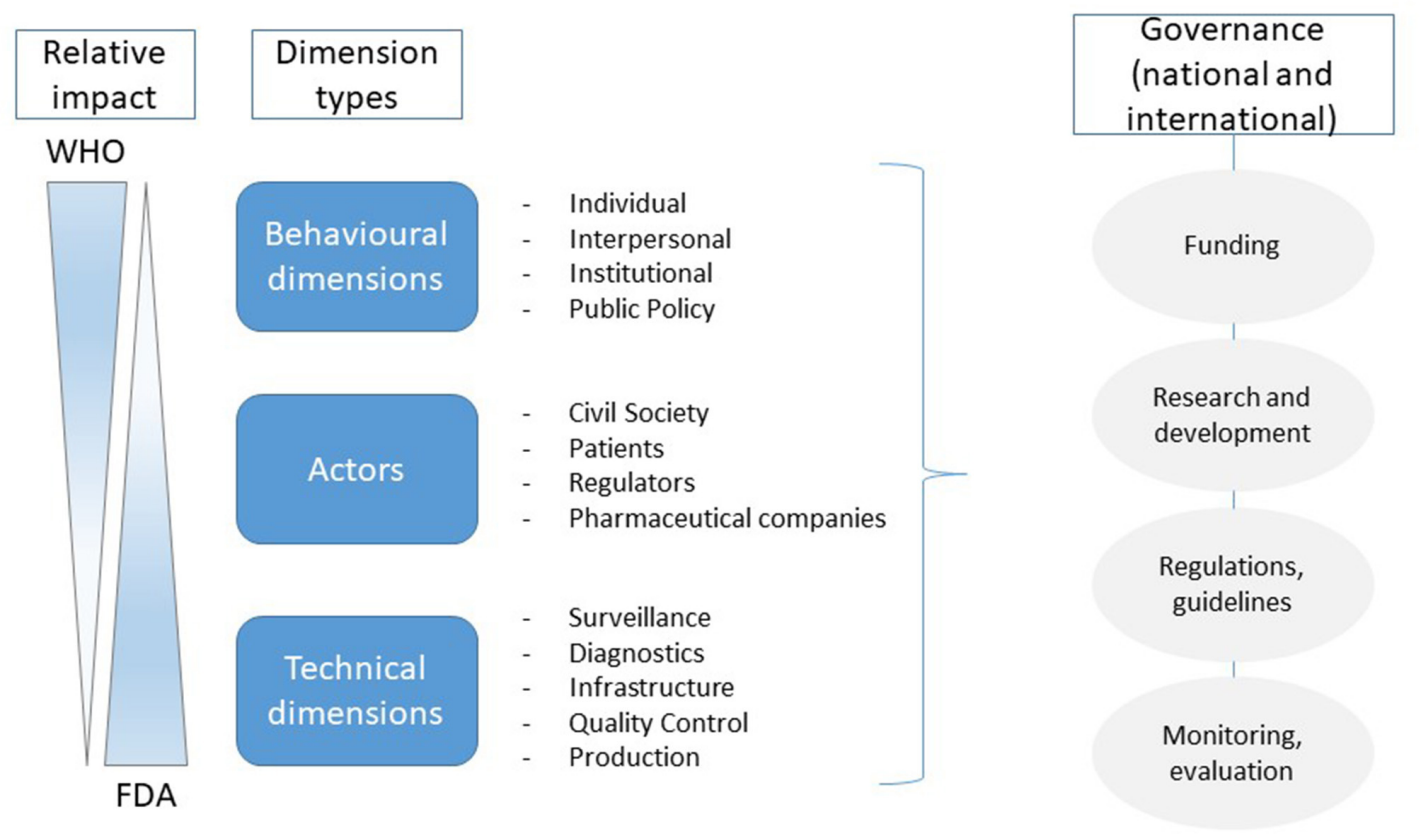
The simplified relative impact of the organizations mentioned in the manuscript (adapted) (49). This schematic representation defines the different influence domains of the main actors during the pandemic and the need for a coordinated international approach for future responses to coronavirus disease 2019 (COVID-19).

It seems inevitable that post COVID-19, there will be a review of policies, guidelines, and regulations relating to individuals' rights and the implementation of drastic public health measures, such as prolonged quarantine measures as well as the governance of new technologically driven solutions within healthcare (relative impact shown in Figure 1). Compulsory “public health-triggered” powers are currently justified under a common legal and ethical standard, taking into account the risk of the pathogen to the individual and the general population, the incidence rate and transmission mode of the pathogen, the effectiveness of available public health interventions, and the availability and type of clinical treatments. In particular, in emerging crises, such as in the case of COVID-19 when the science is uncertain, the adoption of the “precautionary principle” is reasonable to ensure public safety. It is expected that post COVID-19, a number of these measures will be evaluated on their timing and effectiveness, whether the nature of the measures and their implementation was proportionate to the risk, and whether the legal assessments of the partial scientific evidence were successful (48).

## Development of Communication Technology-Based Approaches

The transformation in healthcare would not be possible if it is not associated with technological innovations in communication, machine learning, and transportation. The expansion of Medicare telehealth coverage amidst the pandemic is a major step in the right direction (50) as well as the increased delivery of healthcare closer to home for chronic neurological patients (51). However, concerns about security remain as not all publicly available tools for videoconferencing comply with internationally accepted standards of confidentiality. This security concern, for example, the phenomenon of Zoom-bombing, applies as much to patients as it does to medical professionals delivering the new remote services. Therefore, technology-empowered approaches must take all necessary steps to safeguard the privacy of their participants.

The silver lining for the post-COVID-19 era is the realization that a significant portion of healthcare activities in the wider sense can be improved by technologically empowered approaches, and some can even be done remotely equally as effectively. For example, for some postoperative follow-ups, phone visits are not necessarily inferior to in-person visits in terms of patient satisfaction, complications, and adverse events (52). Where there needs to be an increased emphasis is the investigation of how technologies can be utilized earlier and/or better in order to provide added flexibility to the responsiveness of the healthcare system in times of crisis. Existing guidelines have supported part of this perspective (53). However, there has been a noticeable struggle to shift the focus of healthcare systems to tackle the current emergency resulting in potential response timelags (54).

## Development of Financial Models to Support Scientific Research, Cooperation, and Crisis Preparedness

COVID-19 led simultaneously to two opposite consequences on laboratory medicine activities. On the one hand, microbiology departments faced a huge increase in their diagnostic activities related to the afflux of COVID-19 suspected patients (55). On the other hand, activities of clinical laboratories not directly related to COVID-19 dropped significantly, including for instance cancer services, which had to adapt to a different, remote-based service model (17, 18). A similar picture was also observed at the institutional/hospital level, with a drop of routine activity (56), and the acute need for reallocation of staff and services (57). Considering these factors, COVID-19 has changed the healthcare business models of basic academic health sciences, public health surveillance, and the industry. The efficient collaboration within informal networks comprising clinical laboratories servicing consortia of hospitals, academic groups, and test manufacturers (forged through previous recent outbreaks and/or operational consolidations) represented a key element in the European response against COVID-19 and in supporting acute clinical and international needs (e.g., utilization of the existing COMBACTE Network) (58).

Thus, it is likely that because of their quick mobilization and response times to the clinical needs, further global activities such as those within the Innovative Medicines Initiative (IMI) framework will be strengthened, hopefully maintaining the breadth of creative approaches. The urgency of the COVID-19 situation forced major healthcare providers to respond often without the ability for a full discussion of the financial costs involved in those emergency responses. However, the scale of investment needed for combatting COVID-19 is certainly ambitious and a key consideration for the immediate future. As such, new public private partnerships are vital, whether this involves drug, vaccine, and/or test development. Unlocking additional financing sources, acknowledging the imperative to link financial returns to the providers of capital, and creating profitable, sustainable financing structures will be central in developing new financial models to support scientific research, cooperation, and crisis preparedness (59).

## Conclusion

The COVID-19 outbreak serves as a reminder that proactive planning for healthcare emergencies as well as an intensified commitment to global public health preparedness remains necessary. The lessons learned on the limitations of extant healthcare systems and their capacity to respond to infectious disease epidemics in the 21st century should be considered, enabling the transformation of future healthcare. In addition, the realization that technologically empowered solutions can be implemented and work well-should constitute the benchmark for the greater integration of such technologies as part of routine healthcare design and provision. Optimal outcomes can be attained where both patients and healthcare providers become active participants in this process. However, for that to be achieved, ethical, regulatory, and legal concerns that emerged during this pandemic need to be addressed. The current global experiences lay the foundation for a significant post-COVID-19 healthcare transformation, so that systems can better prepare to address the next global threat(s) of the 21st century.

## Author Contributions

All authors listed have made a substantial, direct and intellectual contribution to the work, and approved it for publication.

## Conflict of Interest

The authors declare that the research was conducted in the absence of any commercial or financial relationships that could be construed as a potential conflict of interest.
